# Assessment of limited cardiac ultrasound to screen for mitral regurgitation

**DOI:** 10.1186/s44156-025-00099-8

**Published:** 2025-12-22

**Authors:** Divya Velury, Benjamin Koethe, Monica M. Dehn, Eileen Mai, Dikran R. Balian, Brian C. Downey, Arsalan Rafiq, Ayan R. Patel, Benjamin S. Wessler

**Affiliations:** Tufts Medical Center, 800 Washington St, Boston, MA 02111 USA

**Keywords:** Mitral regurgitation, Screening, Point of care ultrasound (POCUS), PLAX imaging, Focused cardiac ultrasound, Pocket ultrasound, Color doppler

## Abstract

**Background:**

Mitral regurgitation (MR) is under-diagnosed and under-treated. Point-of-care ultrasound devices now include color Doppler technologies that can enhance the physical exam. Focused handheld imaging protocols might improve detection of mitral regurgitation.

**Methods:**

Patients were prospectively scanned with a limited protocol using the Butterfly IQ + device at Tufts Medical Center (Boston, MA). Comprehensive staging of MR was done with cart-based transthoracic echocardiography (TTE) during the same encounter. Handheld imaging-based MR severity was assessed by a board certified echocardiographer blinded to the cart-based results. Performance of handheld color Doppler imaging for identifying significant MR was assessed across a range of disease prevalence values.

**Results:**

100 patients were prospectively scanned, 45% were women. The median age was 75 (IQR 70–80). The Median LVEF was 60 (IQR 54–64%). Overall, 44 (44%) had some degree of MR. 8 (8%) had significant MR, defined as ≥ moderate. The sensitivity of handheld parasternal long axis color Doppler imaging for the detection of ≥ moderate MR was poor (38% 95% CI 8.5% − 75.5%). PPV and NPV ranged from 25% to 60% and 91.9%-98.9%, across a range of potential disease prevalence rates. There was poor agreement between handheld ultrasound and TTE derived MR grade (weighted kappa 0.08, 95% CI -0.01–0.17). There was modest correlation between assigned grades by each imaging method (Spearman *r* = 0.41, *p* < 0.001).

**Conclusions:**

Limited handheld color Doppler imaging has limited value in screening for significant mitral regurgitation. Routine assessment of handheld imaging protocols proposed for cardiac disease detection are needed.

**Clinical trial number:**

Not applicable.

## Background

Mitral regurgitation (MR) is among the most prevalent valvular heart diseases and is a significant cause of heart failure if left untreated [1]. The prevalence of significant MR has been noted to be anywhere from 2% to 10% depending upon population studied [1, 2], with a prevalence of ~ 9.3% in age 75 and older. With improvements in the medical, surgical, and percutaneous management of both primary and secondary MR, mortality associated with MR is now decreasing [3]. Unfortunately, MR is often detected at a late stage and only after adverse cardiac remodeling or symptoms develop. Further, lack of intervention is associated with high mortality [4]. Since cardiac auscultation has poor sensitivity and specificity for detecting valvular heart disease [5, 6], there is now significant interest in developing new approaches to diagnose MR.

Focused Cardiac Ultrasound (FCU) from handheld ultrasound devices is an appealing approach to diagnosing structural heart disease. These new devices have been proposed as tools to augment the physical exam and enable detection of structural heart disease upstream of traditional echocardiogram laboratories [7]. Modern devices now include capabilities for color Doppler imaging that can identify regurgitant valve lesions. However, these technologic innovations have not been rigorously tested for evaluation of valve disease. Here we assess a limited handheld imaging protocol that includes color Doppler imaging as a method to screen for significant mitral regurgitation (MR).

## Methods

This is a prospective study of adults ≥ 65 years of age referred for comprehensive transthoracic echocardiography (TTE) at Tufts Medical Center (Boston, MA). Since the prevalence of MR increases with age, we identified this population to enrich for the disease of interest and to more closely mirror an appropriate screening population. Participants were approached at the time of routinely scheduled TTE for participation.

### Comprehensive protocol

Comprehensive TTE imaging was done as part of routine care. These images were obtained on a cart-based machine in accordance with standard protocols by a registered cardiac sonographer.

### Focused protocol

Parasternal long axis (PLAX) imaging with and without color Doppler was obtained by trained sonographers using the Butterfly IQ + device during the same encounter. The Nyquist limit was maintained at the default for cardiac imaging on this device (59.8 cm/s). This limited protocol was selected as it is a scalable protocol that can be taught to non-imaging experts.

### MR analysis

Ground truth severity of MR was extracted from the formal read of TTE done during the study encounter. MR was graded in standard integrative fashion [8]. Handheld imaging was reviewed in a blinded manner by a board certified echocardiographer. The severity of MR on focused imaging from the handheld device was assessed based upon the mitral valve morphology and the MR jet visualized on color Doppler imaging. In standard fashion, MR was graded as none (0), trace (1), mild (2), moderate (3), and severe (4).

### Statistical analysis

Sensitivity/specificity for detecting significant (≥ moderate MR) as well as the percentage of ≥ moderate MR cases that would be missed using this protocol are reported. Correlation between the handheld imaging grade of mitral regurgitation and the TTE was evaluated utilizing Pearson’s correlation. Lastly, we model the PPV/NPV across a range of disease prevalence rates. Referrable MR was defined as MR that appeared ≥ moderate MR on focused imaging.

## Results

### Patients

100 patients were scanned using the Butterfly IQ + device (Table 1). Median age was 75 (IQR 70–80) years. Median left ventricular ejection fraction was 60% (IQR 54.3–63.8%). These patients are representative of patients referred for comprehensive TTE imaging.

**Table 1:** Patient characteristics

*N* = 100		
Patient Characteristics	Value	IQR
Age (median)	75	70–80
**Sex (n)**		
M	55	
F	45	
LVEF (median)	60	54–64
HTN (n)	81	
Smoking (n)	57	
Diabetes (n)	26	
Myocardial Infarct (n)	15	
**Aortic Stenosis**		
None	80	
Mild	3	
Mild to moderate	3	
Moderate	5	
Moderate to severe	6	
Severe	3	
**Tricuspid Regurgitation (n)**		
None	4	
Trace	51	
Mild	34	
Mild to moderate	6	
Moderate	2	
Severe	3	
**Right Ventricular Function (n)**		
Normal	85	
Low normal	9	
Mildly reduced	3	
Moderately reduced	2	
Severely reduced	1	
Left Atrium Volume Indexed (ml/m2) (median)	36.6	27.6–46.2

### Ground truth

44 (44%) of patients had some degree of MR. Eight patients (8%) had ≥ moderate MR. Seven (87.5%) of the significant MR cases were from a primary mechanism.

### Handheld imaging

All handheld imaging was interpretable for MR severity. Some degree of MR defined as ≥ 2 (mild) was identified in 7 (7%) of patients. Significant MR, defined as ≥ 3, was identified in 6 (6%) of cases. Five cases (62.5%) of patients with ≥ moderate MR as determined on cart-based imaging were graded as none-mild on handheld imaging.

### Handheld imaging as a screening test

Sensitivity of this handheld ultrasound imaging technique for detecting any MR (defined as mild or greater) was 11.4% (95% CI 3.8%-24.6%). Sensitivity for detecting significant MR (defined as moderate or greater) was 38% (95% CI 8.5% − 75.5%) and specificity was 96% (95% CI 90.7%-99.3%) (Table 2).

**Table 2:** Confusion matrices for the detection of mitral regurgitation utilizing the butterfly IQ + Device

Moderate or greater on TTE, graded as 3 (moderate)	MR	No MR	
Test +	3	3	6
Test -	5	89	94
	8	92	
	SN = 38% (CI 8.5–75.5%)	SP = 96% (CI 90.7–99.3%)	

### Agreement between cart-based and handheld MR assessment

There was poor agreement between handheld ultrasound and TTE derived MR grade (weighted kappa 0.08, 95% CI -0.01–0.17) (Table 3). There was modest correlation between assigned grades by each imaging method (Spearman *r* = 0.41, *p* < 0.001).

**Table 3:** Kappa statistics reveal a poor agreement between TTE and POCUS grading

Kappa Statistics				
Statistic	Estimate	Standard error	95% confidence limits	
Weighted Kappa	0.0798	0.0456	-0.0095	0.1690

### Modeling screening environments

To assess this method of screening for MR, we studied test performance across a range of disease prevalence (Table 4). At disease prevalence that matches the prevalence of moderate MR for patients age ≥ 70 (~ 2%), the PPV was 25% (CI 5.3% -66.4%) with a NPV of 98.9% (CI 89.5%-99.7%). In a high-risk MR-enriched population (8% ≥ moderate MR prevalence), the PPV and NPV were 50% (95% CI 19.3%-80.7%) and 94.7 (95% CI 91.2%-96.8%) respectively.

**Table 4:** Performance of FCU for detecting significant MR as disease prevalence varies

Prevalence	Sensitivity (CI)	Specificity (CI)	PPV (CI)	NPV (CI)
~ 2%	50% (1.3–98.7%)	96.7% (90.8–99.3%)	25% (5.3–66.4%)	98.9% (89.5–99.7%)
~ 4%	50% (6.8–93.4%)	96.7% (90.8–99.3%)	40% (13.1–74.6%)	97.8% (94.3–99.2%)
8%	37.5% (8.5–75%)	96.7% (90.8–99.3%)	50% (19.3–80.7%)	94.7% (91.2–96.8%)
12%	37.5% (8.5–75.5%)	96.6% (88.3–99.6%)	60 (22.7–88.4%)	91.9 (86.9–95.1%)

## Discussion

**Fig. 1:**
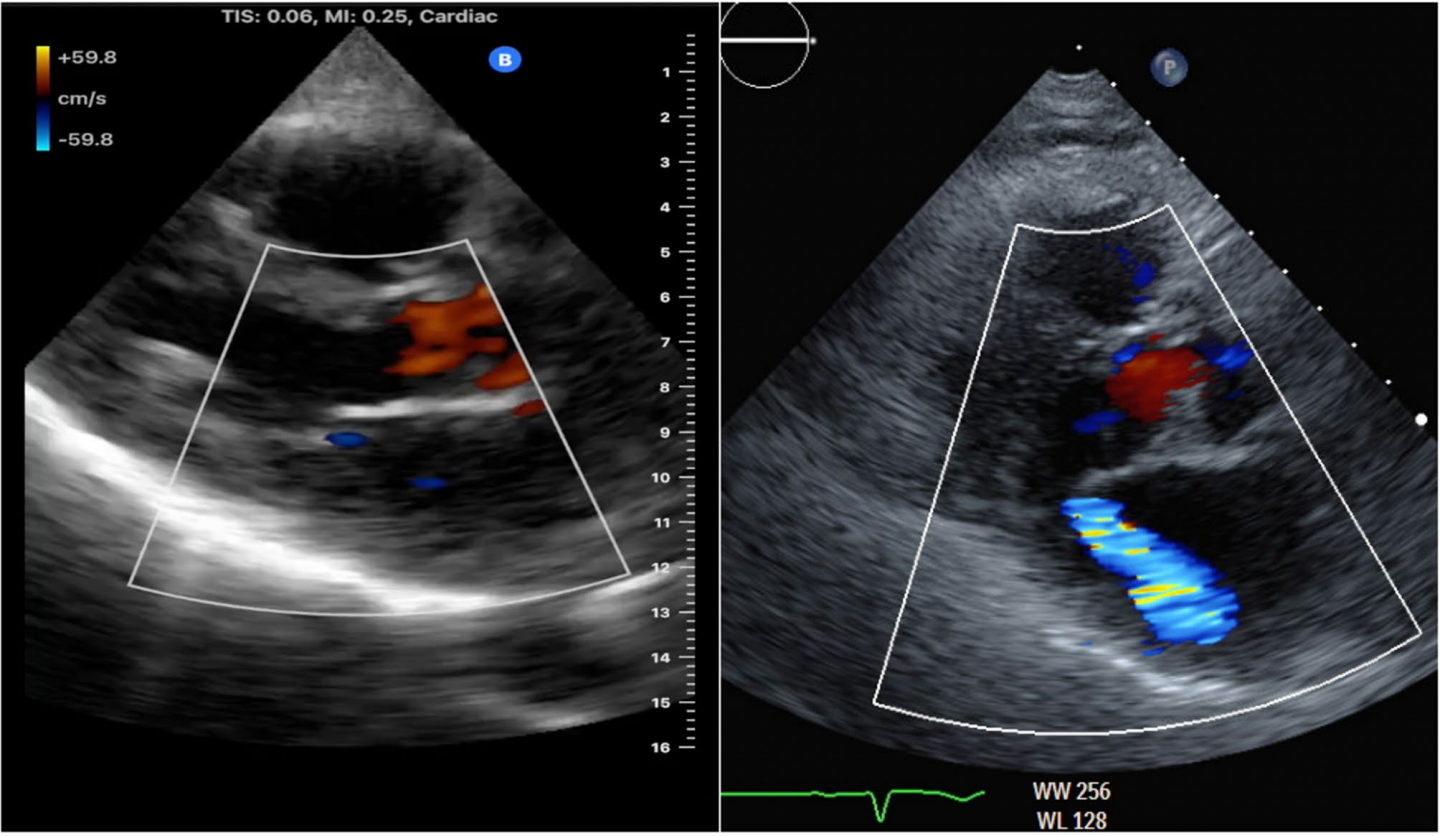
Comparison of a significant MR imaged on the Butterfly IQ+ (left) vs. cart-based Echocardiography (right)

The primary finding from this study is that focused color Doppler imaging with a handheld ultrasound device has limited accuracy to identify patients with significant MR (Fig. 1). This protocol missed a significant number of patients with MR that should be referred for comprehensive imaging. While more comprehensive handheld cardiac imaging protocols might enable accurate disease detection, this report highlights the need to comprehensively evaluate new imaging technologies and protocols before they are deployed for cardiac disease detection.

There is substantial interest in identifying patients with structural heart disease from limited cardiac ultrasound imaging [9, 10]. Handheld ultrasound is an appealing method of disease detection because it can be performed by non-experts and deployed upstream of traditional echocardiogram laboratories. FCU has been proposed as a method of disease detection and when combined with physical examination, FCU might improve the detection of significant MR [11, 12]. Limited imaging protocols with 1–2 views are particularly attractive because they can be easily taught to non-physicians and providers in specialties other than cardiology; however, there are major concerns about failing to identify patients who should be referred for comprehensive TTE because of ‘normal’ focused imaging [13]. Our study validates these concerns and shows that this simple single view handheld protocol is not an effective protocol for screening for significant MR.

Guidelines for assessment of MR rely on multiple methods including quantitative measurements such as proximal isovelocity surface area (PISA)–based effective regurgitant orifice area (EROA) and regurgitant volume, color Doppler jet/LA area, pulmonary vein flow, peak transmitral E wave [14]. Most of these variables are reserved for diagnostic quality cart-based imaging, and there are open questions about whether regurgitant lesions can be accurately assessed upstream of the echo lab. While the device used in this study has capabilities for M mode and some adjustments in color gain, advanced measurements are not be accessible to most non-experts. Imaging protocols for earlier disease detection need to balance accessibility and accuracy. Our study employed a limited handheld protocol because it is scalable and has been previously proposed as a method for disease screening [9]. We found that even when this imaging was performed by certified sonographers, this protocol could not reliably identify patients with significant MR who should be referred for more comprehensive imaging. While it is possible that more extensive imaging protocols that include apical windows might yield different results [7], the scalability of more extensive protocols is less certain and the quality of imaging from non-experts remains unknown.

We used moderate MR as the “referrable threshold” since it is necessary to identify cases earlier in disease course to allow for appropriate longitudinal follow-up [14]. In our protocol in a population enriched for MR, >50% of patients with significant MR would not be referred for additional testing. This raises concerns about apparently ‘normal’ handheld imaging that might be used to ‘rule out’ significant cardiac pathology. As shown in Table 4, performance of this screening protocol is heavily dependent on disease prevalence where low disease prevalence substantially limits the value of screening. When disease prevalence is significantly enriched, apparent performance of this screening method (as assessed with PPV and NPV) appears to increase. This modeling study reinforces the importance of identifying appropriate populations for screening. A kappa coefficient of 0.08 indicates poor MR categorical agreement between the point of care ultrasound and TTE; however, the Spearman correlation showed modest correlation between limited handheld imaging assessment and TTE ground truth MR grading. It appears that a more comprehensive handheld ultrasound protocol is needed to reliably identify significant MR, recognizing this will increase the complexity of image acquisition.

There are a few limitations to this study. Handheld ultrasound imaging in this study was done by trained sonographers. Therefore, this study represents the ‘best case scenario’ for this limited imaging protocol and device. It is likely that color Doppler imaging by non-experts will have even lower sensitivity for the detection of significant MR depending upon imaging quality. Additionally, this study was done on a convenience sample already referred for TTE. Future protocols should be tested on a truly asymptomatic population upstream of the echocardiography laboratory. Prior studies assessing ability to detect MR via handheld devices have had some success utilizing non expert imaging with specific training protocols [5]. However, these studies differ by utilization of a different device, incorporation of multiple views (including parasternal long and apical views), and incorporation of history/physical exam [5, 15]. More extensive imaging protocols might enable better MR detection at the cost of scalability [5, 11, 15]. More recently, artificial intelligence methods have been developed to help interpret ultrasound images. Early reports that these tools might enable detection of MR using limited imaging [16] are promising, though have only been studied on diagnostic quality echocardiograms and have not yet been evaluated using imaging done by non-experts upstream of the echocardiogram laboratory.

## Conclusions

This limited cardiac imaging protocol has limited sensitivity for detecting significant mitral regurgitation. This study demonstrates the need for rigorous assessment of focused cardiac ultrasound protocols for upstream detection of significant cardiac pathology prior to their use as potential screening tools.

## Data Availability

No datasets were generated or analysed during the current study.
